# Cardiac magnetic resonance imaging (CMR) characteristics in apical versus non-apical hypertrophic cardiomyopathy (HCM)

**DOI:** 10.1186/1532-429X-18-S1-P269

**Published:** 2016-01-27

**Authors:** Amardeep Ghosh Dastidar, Priyanka Singhal, Giuseppe Venuti, Antonio Matteo Amadu, Anna Baritussio, Alessandra Scatteia, Estefania De Garate, Chris B Lawton, Jonathan C Rodrigues, Chiara Bucciarelli-Ducci

**Affiliations:** grid.410421.20000000403807336NIHR Cardiovascular Biomedical Research Unit, Bristol Heart Institute, Bristol, United Kingdom

## Background

Hypertrophic cardiomyopathy (HCM) is the most common genetic cardiac disorder, and the most common cause of sudden cardiac death (SCD) in young adults. The 3 main phenotypes are asymmetric, concentric or apical, with asymmetric being the most common. Literature suggests apical HCM to be a rare variant (variable prevalence) with better prognosis but the data is limited.

## Aims

Provide a contemporary prevalence and characteristics of apical HCM in a large tertiary clinical CMR service.

## Methods

Approximately 3,100 CMR scans were reviewed from our CMR registry (Jan 2014 to Mar 2015). comprehensive CMR protocol was used including cines, early and late gadolinium enhancement imaging. 114 consecutive HCM patients were identified. A Asymmetric HCM was defined as: septal to free wall thickness ratio of > 1.3; apical HCM as apical wall thickness of > 15 mm or apical to basal LV wall thicknesses ≥ 1.3-1.5; and concentric HCM as symmetrical hypertrophy of ventricular wall without any regional preferences. Non-apical HCM group (comprising of asymmetric and concentric phenotypes) were compared with apical HCM. Fisher's exact t-test and unpaired t-test were performed for statistical significance. P-value < 0.05 was statistically significant. Univariate and multivariate logistic regression analyses were performed to determine the CMR predictors of apical HCM.

## Results

The final study sample consisted of 104 patients with HCM with median age 60years (IQR = 54-70) and 70% male, (10 patients excluded due to uncertain diagnosis) 70% non-apical HCM; the remainder 30% apical HCM. In the non-apical HCM group, 5 patients had concentric HCM and the rest had asymmetric HCM. The. The mean maximum LV wall thickness, mean indexed LV mass, mean indexed stroke volume, prevalence of LVOTO and SAM were significantly greater in non-apical group. Table 1 The presence of LGE was high in both groups (>85%) and was not statistically different. The univariate predictors of apical HCM included maximum LV wall thickness, indexed stroke volume, LVOT obstruction whereas in the multivariate model maximum LV wall thickness remained the only significant predictor.

**Table 1:** CMR characteristics of Apical vs non-Apical HCM

CMR findings:	Total Cohort (n=104)	Non-apical (n=73)	Apical (n=31)	P-value
Mean LVEF (%)	69.7	68.4	72.6	0.0552
Mean LVEDVI (mL m-2)	73.7	76.7	66.8	0.0718
Mean LVESVI (mL m-2)	23.8	25.5	20.1	0.1177
Mean indexed stroke volume	53.1	55.9	46.4	0.0333
Mean max. LV wall thickness (mm)	18.2	19.3	15.6	0.0001
Mean indexed LV mass	93.5	98.4	82.4	0.0102
LVOTO	35.2	41.1	12.5	0.0403
SAM	31.4	38.9	6.25	0.0143
LGE %	86.9	85.7	89.7	0.8063

LVEF, left ventricular ejection fraction; LVEDVI, left ventricular end diastolic volume index; LVESVI, left ventricular end systolic volume index; LVOTO, Left ventricular outflow tract obstruction; SAM, systolic anterior valve motion; LGE, late gadolinium enhancement.

## Conclusions

Our study suggests that in the era of CMR, the prevalence of apical HCM to be almost 1/3^rd^ of all observed HCM cases. The study also demonstrates that the prevalence of LGE was high also in the apical HCM group suggesting that the better prognosis that apical HCM is thought to have based on the absence of myocardial fibrosis should be reconsidered. Further large prospective multi-centre trials are needed to establish the key differences thereby understanding the pathophysiology.

